# Modified differential lysis for sexual assault samples using a combined enzymatic and alkaline approach

**DOI:** 10.1093/fsr/owae022

**Published:** 2024-04-02

**Authors:** Brittany C Hudson, Tracey Dawson Green

**Affiliations:** Department of Forensic Science, Virginia Commonwealth University, Richmond, VA, USA; Integrative Life Sciences, Virginia Commonwealth University, Richmond, VA, USA; Department of Forensic Science, Virginia Commonwealth University, Richmond, VA, USA

**Keywords:** forensic DNA analysis, DNA mixtures, sexual assault, differential lysis, prepGEM™, alkaline

## Abstract

Sexual assault sample processing, despite recent funding and research efforts, remains time-consuming, labourious, and inefficient. These limitations, combined with the prevalence of sexual assaults, have prompted the need to develop a cheaper, quicker, and more robust method for separating victim and perpetrator contributions within sexual assault evidence so that analysts can keep pace with submissions and cases can be resolved in a timely manner. Thus, this study examined the use of a combined enzymatic and alkaline approach for differential cell lysis—with the goal of developing a quick, cheap, and more efficient DNA isolation method. Quantification results for this assay revealed that (72.0 ± 18.3)%, (15.8 ± 14.2)%, and (29.5 ±  23.7)% of total DNA were retained in sperm fractions for neat semen, neat vaginal, and semen–vaginal mixture eluates, respectively. Short tandem repeat (STR) analysis of mixture samples processed with this technique exhibited sperm fraction DNA profiles with mean male-to-female ratios of 1.74:1, which was a 3.01 ± 2.30-fold improvement in male-to-female ratios and led to the recovery of 5.90 ± 7.80 unshared male contributor alleles in sperm fractions that were otherwise undetected in unseparated controls. Overall, this study presented a modified differential lysis approach using *prep*GEM™ and sodium hydroxide treatments that can accomplish cell elution and fractional lysis within 25 min. Future studies should investigate alternative “non-sperm” cell lysis methods to enhance lysis efficiency and minimize the potential for inhibition, as well as the optimization and automation of this technique.

**Key points:**

- Traditional sexual assault sample processing methods are time-consuming and inefficient.
- This modified differential lysis method produces lysates with sufficient DNA yield and quality.
- A combined technique using enzymatic and alkaline lysis can accomplish fractional separation.
- Lysis with *prep*GEM and NaOH absent purification is compatible with downstream processes.

## Introduction

Despite many technological and sensitivity improvements in forensic DNA analysis, the processing of sexual assault samples remains time-consuming and inefficient. This, combined with the fact that ~22% of violent crimes reported in the USA in 2021 were comprised of rape or sexual assault [1], has led to an ongoing sexual assault evidence collection kit (SAECK) backlog. Further, despite nationwide efforts to reduce such backlogs, recent legislation that requires the submission and processing of all collected SAECKs is compounding this issue, necessitating the development of sample processing techniques that are cheaper, quicker, and better at recovering and isolating DNA in order to offset the demands on laboratories.

Differential lysis has been the most widely accepted and traditionally used technique for separating the predominant cell types found in sexual assault samples to date. This method, which was originally described by Gill et al. [2] in 1985, takes advantage of the difference in lysis susceptibility between non-sperm and sperm cells when exposed to certain reagents in order to accomplish fractional separation of victim and perpetrator DNA contributions. Ideally, this technique would culminate in a sperm fraction containing only DNA from the male (which may be a consensual partner and/or the perpetrator), as well as a non-sperm fraction containing DNA from the lysed female vaginal epithelial cells (and male epithelial cells). However, traditional differential extraction often inefficiently isolates sperm and non-sperm cells due to many factors, such as the presence of old or degraded sperm cells that are susceptible to premature lysis, excess female epithelial cells that fail to completely lyse and remain within the sperm fraction, loss of sperm due to repeated wash steps, as well as poor and tedious manual pipetting technique [3, 4]. Further, traditional differential lysis requires long incubations, relies heavily on manual pipetting and transfer steps, and is inherently difficult to fully automate.

Given these drawbacks, many modified techniques have been investigated and reported for processing sexual assault samples. Cotton and Fisher [4] provided a summary of several modified techniques that have been explored, focusing on those that have attempted to reduce incubation times and minimize female DNA carryover. Some of the earliest modifications involved simple lysis condition adjustments, such as milder reagents and increased temperature, to avoid the unintended loss of sperm DNA and promote more efficient lysis of epithelial cells [5, 6]. A second mild lysis step prior to sperm cell lysis has reportedly improved male-to-female (M:F) DNA ratios in sperm fractions by as much as 6-fold [7] and reduced non-sperm DNA carryover by 5.5-fold without concomitantly reducing sperm DNA recovery [8]. In addition, some studies have focused on the replacement of dithiothreitol (DTT) for sperm lysis with Tris(2-carboxyethyl)phosphine (TCEP) or 1-thioglycerol in an attempt to decrease incubation times [9–12] and to avoid the effects of DTT on downstream processes if not removed *via* purification (i.e., for direct-to-amplification applications) [10–13]. Unfortunately, despite the reduced time and cost requirements, many of these techniques still result in mixtures and/or lead to incomplete male DNA profiles in sperm fractions. In addition, these methods typically require a post-lysis purification step—which can lead to additional loss of DNA and add time to the forensic DNA workflow—as well as hands-on processing that includes several tube transfers.

In order to drastically reduce sample processing times and costs, as well as minimize DNA loss, newer lysis methods that omit subsequent DNA purification of the lysate have been developed. These methods often utilize detergents and/or enzymes to break open membranes and denature or degrade proteins. One example of this approach is the *prep*GEM™ kit from MicroGEM (Charlottesville, VA, USA), which implements the thermophilic enzyme EA1 to accomplish cell lysis at 75°C in mere minutes. Many studies have applied this kit to forensically relevant samples and have obtained usable DNA profiles without the need to further purify the resulting lysates [14–20]. While this method works for non-sperm cells, additional techniques have been reported for more robust cells, such as sperm. *forensic*GEM™ Sperm (MicroGEM) utilizes an additional enzyme cocktail known as *Acrosolv* to accomplish sperm cell lysis at a lower temperature, followed by EA1 lysis to further degrade proteins as well as the *Acrosolv* itself [12, 21, 22]. On the other hand, additional sperm lysis techniques that do not require enzymes have also been explored. Notably, Schellhammer et al. [12] investigated several direct-to-amplification techniques for sperm lysis involving commercial, “homebrew” and “natural decondensation” reagents. Results from this study found several candidate methods for this application that could produce usable STR profiles with reduced time, cost, and volumes; however, the authors ultimately recommended a 5-min incubation with sodium hydroxide at 75°C, followed by neutralization with Tris–HCl [12]. Not only could these direct-to-amplification methods reduce overall time and costs associated with processing sexual assault samples *via* traditional bench methodology (e.g., tube transfers, spin baskets, and so on), but they are also much more likely than traditional DNA extraction and purification to be compatible with automation and microfluidic platforms [15–18, 23–26].

Thus, the research herein attempted to address and overcome the previously outlined drawbacks of traditional differential lysis by exploring a consecutive enzymatic (*prep*GEM™) and alkaline approach. Use of these direct-to-amplification techniques, even in a traditional microcentrifuge tube environment, would ideally increase sample processing efficiency by more effectively lysing non-sperm cells, reducing non-sperm DNA carryover, retaining as many sperm cells as possible within the sperm fraction, as well as providing time and cost savings. Further, the development of such a technique could bring the field closer towards a more fully automatable method for sexual assault sample processing.

## Materials and methods

### Sample collection and preparation

Semen samples and vaginal swabs were collected from 10 anonymous donors respectively with written informed consent (*via* self-collection) in accordance with the Virginia Commonwealth University-approved Institutional Review Board (IRB) protocol HM20002931. Semen was diluted 1:60 by volume using Gibco™ 1X Dulbecco’s phosphate-buffered saline (DPBS) (Fisher Scientific; Waltham, MA, USA). Cells were eluted from vaginal swabs by submerging a half-swab cutting in 200 μL DPBS and incubating at 37°C for 2 h, with brief vortexing every 15 min. All dilutions and eluates were stored at 4°C.

Semen (*n* = 10), vaginal (*n* = 10), and semen–vaginal mixture (*n* = 10) samples were dried onto Fisherbrand™ PurSwab foam swabs (Fisher Scientific) prior to subsequent testing. All samples were prepared as indicated below in tubes. Subsequently, a single foam swab was dipped into each tube and allowed to absorb the entire sample. This process was repeated to generate several swabs for each vaginal and semen donor, as well as each unique vaginal–semen mixture. For semen swabs, 30 μL of 1:60 semen were combined with 50 μL of DPBS. Vaginal swabs were prepared similarly by combining 30 μL of DPBS and 50 μL of vaginal eluate. Mixtures were prepared by combining 30 μL of 1:60 semen with 50 μL of vaginal eluate. When processed in previous experiments without any fractional separation, this specific mixture preparation method has generated approximate mean 1:1 M:F ratios in resulting STR profiles within our lab (data not shown). For each sample, multiple swabs were prepared to accommodate all testing. Swabs were allowed to absorb the samples prior to drying overnight at room temperature. Once dry, swabs were cut into fourths and stored at 4°C until testing; all swabs were tested within 2 months of preparation.

All subsequent testing utilized one-fourth of a foam swab, which should theoretically contain ~6 000 to 19 000 sperm cells based on the average sperm counts in normal semen and the dilutions used herein [27–32]. Samples were eluted from swab cuttings by combining cuttings with 20 μL of DPBS in a new tube and incubating at room temperature for 5 min, with brief vortexing every minute. Semen, vaginal, and mixture samples were subjected to differential cell lysis for this study (as described below); however, additional swab cuttings of each mixture sample (*n* = 10) were not subjected to differential lysis (i.e. “unseparated” mixtures) and were assessed as unseparated controls.

### Differential cell lysis

**Figure 1:**
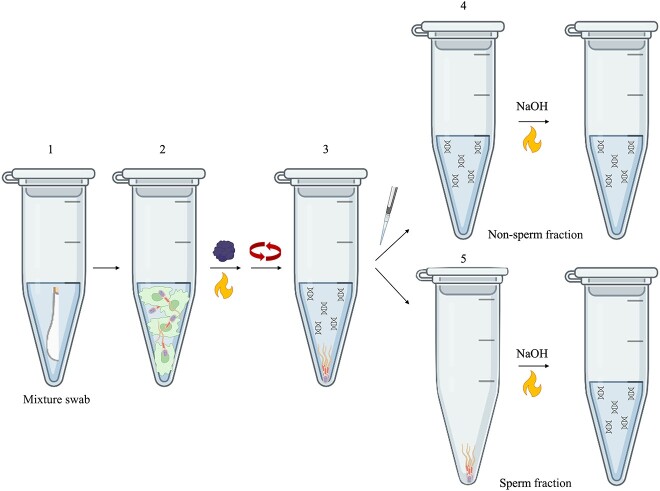
Overview of the modified differential lysis technique using a combined enzymatic and alkaline approach. Cells are first eluted from a swab cutting (1). Enzymatic lysis of non-sperm cells is conducted using *prep*GEM™ (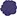) and heat (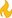) (2), then intact sperm cells are pelleted *via* centrifugation (3). The entire supernatant is removed as the non-sperm fraction (4), leaving behind the pellet as the sperm fraction (5). Both non-sperm and sperm fractions are then subjected to alkaline solution at 75°C to lyse all remaining cells (figure created in part using BioRender.com).

Semen, vaginal, and mixture sample eluates (~19 μL) were transferred to 0.2 mL PCR tubes prior to differential cell lysis. Enzymatic lysis of non-sperm cells was then performed using the *prep*GEM™ Universal kit (*prep*GEM; MicroGEM) (Figure 1). To accomplish this, 0.5 μL *prep*GEM™ enzyme, 5.0 μL 10X Blue buffer, and 25.5 μL HyPure Molecular Biology Grade Water (MBG H_2_O; GE Healthcare Life Sciences, Chicago, IL, USA) were added to each sample. Samples were then incubated using the ProFlex™ 3 × 32-well PCR System (Applied Biosystems™ Foster City, CA, USA) as follows: 75°C for 5 min, then 95°C for 2 min. A subsequent test of this method using the 2X *prep*GEM™ enzyme (i.e. 1.0 μL instead of 0.5 μL) for non-sperm lysis was conducted, keeping all other steps and conditions the same.

Following non-sperm lysis, samples were centrifuged at 17 000 *× g* for 5 min to pellet intact sperm cells. The entire supernatant (50 μL) was then removed as the non-sperm fraction, and the sperm pellet was resuspended in 50 μL MBG H_2_O. Alkaline lysis was performed on non-sperm and sperm fractions according to the method described by Schellhammer et al. [12] (Figure 1).

Additional samples of each mixture created (*n* = 10) were processed alongside differentially lysed samples to serve as an untreated, unseparated control for this technique. For this, the previous procedure was performed, but the removal of the non-sperm fraction after *prep*GEM lysis was omitted (i.e., no fractional separation occurred).

### DNA quantification

Human and male DNA quantities within all resulting lysates were determined using the Investigator® Quantiplex HYres kit (QIAGEN; Hilden, Germany) on the Applied Biosystems® 7500 Real-Time PCR System (Thermo Fisher Scientific; Waltham, MA, USA). Manufacturer recommendations were followed [33], with modifications for half-volume reactions. Thus, 4.5 μL Reaction Mix, 4.5 μL Primer Mix IC YQ, and 1.0 μL template DNA were combined in each well. DNA standards and no template controls were also processed alongside the samples. The resulting data were analysed using Sequence Detection System (SDS) software v1.4 (Applied Biosystems™), with automatic baseline and threshold settings for each target.

To identify any possible signs of inhibition, an assessment of qualitative metrics in the resulting amplification and component plots was conducted as previously described by Hudson et al. [13]. Additionally, several quantitative metrics were assessed. Total and fractional human DNA yields for each sample were calculated by multiplying the human target’s concentration by the sample volume; this was repeated for the male target. To determine the percentage of human and male DNA in each fraction, the fractional DNA yield was divided by the total DNA yield (i.e., the sum of DNA yields in sperm and non-sperm fractions) and multiplied by 100. The mean and standard deviation for each experimental group were then calculated, and all comparisons were assessed using a Student’s *t*-test (*α* = 0.05). Given the reported proportions of sperm (~88%) and non-sperm (~12%) cells within normal semen, and upon correcting for ploidy, the theoretically expected percentage of seminal DNA originating from sperm cells is 80% [4, 27, 28, 34]; these values were used to gauge the ability of the technique described herein to retain sperm DNA within sperm fractions for semen. M:F DNA ratios in unseparated controls, sperm fractions, and non-sperm fractions from processed mixture samples were calculated by dividing the male DNA concentration by the difference between the human and male DNA concentrations. These values were then averaged for each experimental group to determine the mean M:F ratio, and a Student’s *t*-test was conducted to determine statistical significance (α = 0.05).

### STR amplification

All samples were amplified using the Promega™ PowerPlex® Fusion 6C System with a template DNA input of 0.25 ng following manufacturer recommendations, but with half-volume reactions; each reaction included 5.0 μL sample (at 0.05 ng/μL), 2.5 μL PowerPlex® Fusion 5X Master Mix, 2.5 μL PowerPlex® Fusion 5X Primer Pair Mix, and 2.5 μL amplification-grade water. In addition, positive and negative controls were amplified alongside the samples. Thermal cycling was conducted on the ProFlex™ 3 × 32-well PCR System following manufacturer-recommended parameters for Fusion 6C [35].

### Capillary electrophoresis and data analysis

Resulting STR amplicons were separated using an Applied Biosystems™ 3500 Genetic Analyser and Data Collection software v4 (Thermo Fisher Scientific) following manufacturer recommendations. One microliter of sample or allelic ladder was combined with 0.5 μL WEN ILS 500 (Promega™) and 9.5 μL Hi-Di™ Formamide (Thermo Fisher Scientific) in each well on the plate. Injection parameters also followed manufacturer recommendations and included a 36-cm capillary array (Thermo Fisher Scientific), POP-4® polymer (Thermo Fisher Scientific), and a 1.2 kV 15 s injection. The resulting STR profiles were analysed with GeneMapper™ *ID-X* software v1.6 (Thermo Fisher Scientific) following manufacturer settings with an analytical threshold of 150 relative fluorescence units (RFU) [35].

M:F ratios within unseparated controls, as well as resulting sperm and non-sperm fractions from processed mixture samples, were calculated by dividing the total peak height for male alleles by the total peak height for female alleles at each locus where there was no allele sharing between donors. These ratios were then averaged across all loci within a single sample, as well as across all samples within an experimental group (e.g., all 10 sperm fractions). A Student’s *t*-test was conducted to compare mean M:F ratios across sperm fractions and unseparated controls (*α* = 0.05).

The M:F ratio fold improvement for sperm fractions was then determined by dividing the mean M:F ratio within a sperm fraction by the mean M:F ratio in its associated unseparated control (e.g., M:F for Mixture 1 sperm fraction / M:F for unseparated Mixture 1). The mean fold improvement was then calculated by averaging the M:F fold improvement for all 10 mixture samples. Sperm fractions were additionally assessed for the number of male contributor alleles that were recovered (i.e., detected above analytical threshold) compared to their corresponding unseparated controls; these numbers were then averaged to obtain the mean number of unshared male alleles recovered.

## Results and discussion

### DNA quantification

**Figure 2:**
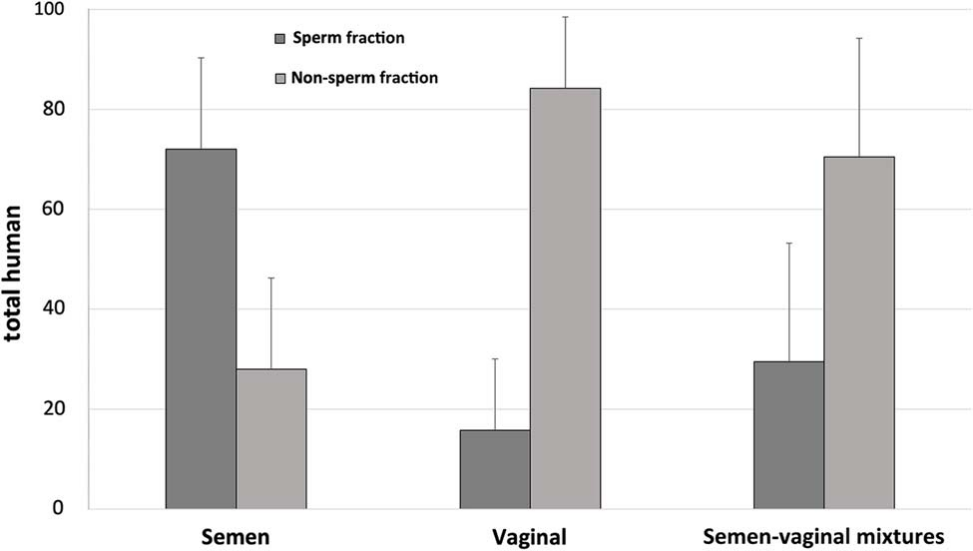
Percentage of total DNA retained in sperm and non-sperm fractions after treatment of semen, vaginal, and mixture eluates with the *prep*GEM/alkaline assay (*n* = 10). This technique was able to retain 72.0% ± 18.3% and 29.5% ±  23.7% of total DNA in sperm fractions from semen samples and mixture samples, respectively.

Evaluation of single-source semen samples revealed that 72.0% ± 18.3% of total DNA was retained in sperm fractions (Figure 2), which is close to the theoretical expectation that 80% of total DNA within normal semen stems from sperm cells [4, 27, 28, 34]. In addition, 15.8% ± 14.2% of total DNA was retained in “sperm fractions” for single-source vaginal samples (Figure 2). Further, although only 29.5% ±  23.7% of the total DNA was retained in sperm fractions of processed mixture samples, this represented 57.3% ±  28.9% of the total male DNA (data not shown). Overall, these results demonstrated the ability of this modified *prep*GEM/alkaline differential lysis technique to sufficiently lyse most non-sperm cells while leaving behind intact sperm cells, which can then subsequently be lysed with more stringent techniques.

**Figure 3:**
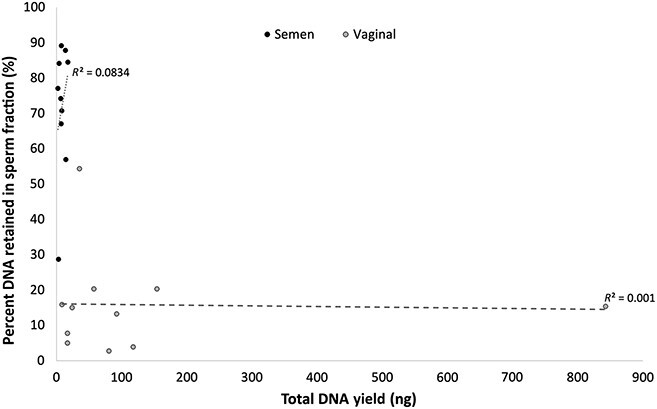
Percentage of total DNA retained in sperm fractions from single-source semen and vaginal eluates relative to the total DNA yield after treatment with the prepGEM/alkaline assay (*n* = 10). Linear trendlines indicated no correlation between cellular input (i.e., total DNA yield) and the percentage of DNA retained in sperm fractions for each sample type, signifying that *prep*GEM can lyse a wide range of cellular input.

The percentage of total DNA retained in sperm fractions for single-source semen and single-source vaginal samples was then plotted against the DNA yield for the entire sample (i.e., the sum of DNA yields in non-sperm and sperm fractions) to determine whether there was a relationship between the percentage of DNA retained within sperm fractions after *prep*GEM/alkaline differential lysis and the cellular input (Figure 3). Ultimately, no association was observed for semen or vaginal samples, as the percentage of DNA retained in sperm fractions for each sample type was consistent regardless of the total DNA (Figure 3). This was promising, as it suggested that *prep*GEM is capable of handling (and lysing) a wide range of non-sperm cells within samples, which is common for sexual assault samples as they are often taken from intimate areas of the victim, and cell count can depend on several factors.

After DNA quantification, M:F ratios were also determined for mixture samples processed with this modified differential lysis technique. Mixture sperm fractions exhibited mean M:F ratios of 1:1.38; this was an improvement over unseparated mixture controls, which exhibited mean M:F ratios of 1:8.02 (Table 1). In fact, the data revealed a 4.4 ±  2.8-fold improvement in the M:F ratios (*P *= 0.26) for sperm fractions when mixtures were subjected to the modified differential lysis technique described herein. Although not statistically significant, this improvement could make a practical and important difference in subsequent STR profiling results since there appears to be an enrichment for male DNA in sperm fractions. Further, mean M:F ratios in non-sperm fractions were 1:16.88, which indicated the ability to transfer female contributions to non-sperm fractions and retain sperm cells within sperm fractions (data not shown). Overall, these results, albeit not statistically significant, demonstrate the feasibility of this method to accomplish differential lysis and provide a foundation for further optimization.

**Table 1:** Male-to-female (M:F) DNA ratios after DNA quantification and short tandem repeat (STR) profiling for semen–vaginal mixture samples (*n* = 10) processed using the *prep*GEM™/alkaline assay either without (i.e., “unseparated”) or with fractional separation.

Sample	DNA quantification		STR profile analysis		M:F fold improvement (SF/unseparated)^*^
	Unseparated	Sperm fraction (SF)	Unseparated	Sperm fraction (SF)	
Mixture 1	1:36.50	1:4.70	1:7.84	1:2.96	2.65
Mixture 2	1:41.70	1:6.20	1:6.20	1:3.45	1.80
Mixture 3	1:13.10	1:3.20	1:2.94	2.30:1	6.75
Mixture 4	1:71.80	1:29.80	1:12.10	1:8.47	1.43
Mixture 5	1:1.80	5.20:10	1.38:1	11.03:1	7.97
Mixture 6	1:13.10	1:6.70	1:3.11	1:1.29	2.42
Mixture 7	1:3.00	1:1.40	2.19:1	1.71:1	0.78
Mixture 8	1:38.50	1:13.80	1:25.75	1:6.27	4.11
Mixture 9	1:17.00	1:3.30	1:5.19	1:2.49	2.08
Mixture 10	1:14.60	1:11.20	1:7.53	1:3.72	2.02
**Average**	**1:8.02**	**1:1.38**	**1:2.01**	**1.74:1**	**3.20 ± 2.40**

^*^ *P* = 0.26.

### STR profiling

**Figure 4:**
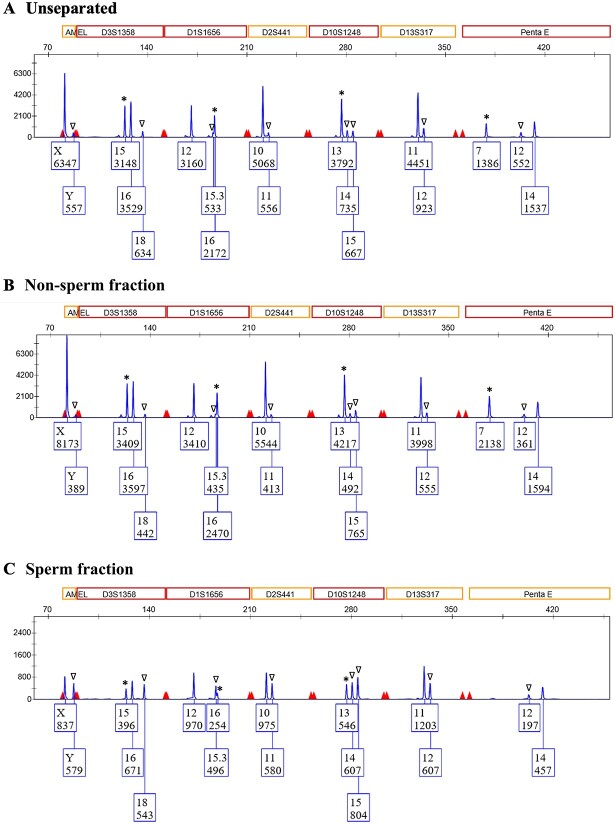
Representative blue channel STR electropherograms for mixtures processed using the *prep*GEM/alkaline differential lysis technique. Unseparated mixture controls exhibited a mean M:F ratio of 1:1.9 (A), while non-sperm fractions had a mean M:F ratio of 1:5.5 (B). Sperm enrichment was demonstrated in sperm fractions, with STR profiles experiencing a 7.97-fold M:F improvement over unseparated controls and a mean M:F of 1.7:1 (C). Asterisks denote unshared female alleles, while unshared male alleles are represented by ∇. Other colour channels demonstrated similar results.

A similar assessment was also performed after STR profiling, as M:F ratios at the DNA quantification step do not always necessarily translate to M:F ratios observed in subsequent STR profiles [36]. Although DNA quantification provides an idea of what to expect within STR profiles, M:F DNA ratios that result from the two processes in the forensic DNA workflow can be slightly different for many reasons, such as different sensitivities between qPCR and STR kits, differences in primers and DNA targets, as well as different buffers and sample input volumes [37, 38]. This evaluation was deemed especially critical for determining the applicability of the modified differential technique described herein, as STR profiles are the final product of the forensic DNA workflow, and therefore, the ultimate efficacy of this technique will depend upon the results obtained at this step. STR profiles of sperm fractions from processed mixtures exhibited mean M:F ratios of 1.74:1, which was a 3.01 ± 2.30-fold improvement (*p* = 0.26) over the mean M:F ratio of 1:1.88 observed in STR profiles for unseparated mixture controls (Table 1, Figure 4). Although not statistically significant, this improvement in M:F ratios (and thus sperm cell enrichment) for resulting sperm fractions demonstrates a practical difference when using this technique. Further, non-sperm fractions exhibited a mean M:F ratio of 1:5.46 (data not shown), indirectly demonstrating the enrichment of male contributions in sperm fractions and highlighting the ability of this technique to generate non-sperm fractions with clear major female contributor profiles. This can be meaningful as it makes profile interpretation easier (i.e., major/minor contributors become clearer) and it provides more confidence in genotyping of the victim DNA profile (serving as an important control for the identification process).

Overall, the resulting STR profiles for sperm fractions demonstrated the ability of a combined enzymatic and alkaline technique to enrich sperm cells. Not only was this apparent when comparing M:F ratios between sperm fractions and their corresponding unseparated controls, but it was also noticeable when considering the number of unshared male contributor alleles that were recovered in sperm fractions but were otherwise undetected in unseparated controls. In fact, 5.90 ± 7.80 unshared male contributor alleles were recovered in sperm fractions, with the most drastic improvement resulting in the recovery of 24 male alleles that were not observed in the unseparated control (i.e., nearly an entire STR profile). Further, the only sperm fractions that failed to recover additional male contributor alleles were samples that already exhibited full male STR profiles within their associated controls. Not only does this highlight the ability of this modified differential lysis technique to enrich for the male contributor within sperm fractions, but it also indicates that this assay could dramatically improve the recovery of male STR profiles from mixture samples with low male contributions and emphasizes the need to assess multiple metrics at STR profiling (other than mean M:F ratios) when determining whether a proposed technique is practically beneficial.

While these results demonstrated the ability of a combined enzymatic and alkaline lysis technique to enrich for sperm within mock sexual assault samples, careful evaluation of sperm fractions also indicated that a portion of intact non-sperm cells may remain in sperm fractions after treatment with *prep*GEM. Thus, additional studies were performed on semen, vaginal, and semen-vaginal mixtures with the 2X *prep*GEM enzyme. Overall, DNA quantification and STR profiling results indicated that non-sperm cell lysis was *not* increased by additional *prep*GEM enzyme; however, premature sperm cell lysis and/or diminished sperm integrity were potential ramifications, leading to reduced sperm cell pelleting as well as sperm loss (data not shown).

## Conclusion

The traditional processing of sexual assault samples remains a very manual, tedious, and inefficient technique that often culminates in complex DNA profile interpretation, fewer case resolutions, and persistent SAECK backlogs. While many studies have attempted to improve this technique, most forensic DNA labs still employ the traditional differential lysis method that was developed in the 1980s—in part because it is already well accepted and validated, but also because newer methods often fail to consistently demonstrate improvements in STR profiling results. Given that two of the primary limitations of the traditional differential method are lysis inefficiency and long incubation times, this study aimed to develop a modified differential cell lysis that could quickly and effectively accomplish both fractional separation and lysis. Further, reduced volumes were implemented to minimize costs and aid in the analysis of lower sperm inputs. Previous studies have demonstrated the ability of enzymatic [14–20] and alkaline [12, 39–41] techniques to effectively lyse cells and obviate the need for DNA purification; thus, we proposed a combined enzymatic and alkaline differential cell lysis assay for processing sexual assault samples.

Overall, this study demonstrated the ability of a combined enzymatic and alkaline lysis technique to differentially lyse non-sperm and sperm cells within forensically relevant samples (*n* = 10). Sperm fractions exhibited enrichment of male contributions and even recovered unshared male contributor alleles—results that could inherently simplify mixture profile deconvolution and lead to additional points of inclusion for statistical analysis. The ability of this method to accomplish cell elution and fractional lysis within 25 min (in comparison to ~170 min when using the QIAamp® DNA Investigator kit for differential lysis and purification [42]) also enables much quicker and more efficient processing of samples, which could help reduce SAECK backlogs. Further, the reduced volumes (~60 μL) and the ability to go directly into STR amplification without further DNA purification make this method much more time- and cost-efficient compared to traditional differential lysis. To further demonstrate and confirm the benefits of the method described herein, future studies should evaluate larger sample sets, replicates, and a more direct comparison to the standard differential lysis technique. Further, because there is evidence of intact non-sperm cells after treatment with *prep*GEM, alternative non-sperm cell lysis techniques should be explored to optimize this assay—with the goals of maximizing non-sperm lysis, minimizing premature sperm lysis, and limiting inhibition. Finally, automation of this technique using centrifugal microfluidics could show promise and further reduce time, variability, and associated costs.
